# Interruption of Wnt Signaling in Müller Cells Ameliorates Ischemia-Induced Retinal Neovascularization

**DOI:** 10.1371/journal.pone.0108454

**Published:** 2014-10-01

**Authors:** Kelu Kevin Zhou, Siribhinya Benyajati, Yun Le, Rui Cheng, Wenbo Zhang, Jian-xing Ma

**Affiliations:** 1 Department of Physiology, Harold Hamm Diabetes Center, the University of Oklahoma Health Sciences Center, Oklahoma City, Oklahoma, United States of America; 2 Department of Medicine Endocrinology, Harold Hamm Diabetes Center, the University of Oklahoma Health Sciences Center, Oklahoma City, Oklahoma, United States of America; 3 Department of Ophthalmology and Visual Sciences, the University of Texas Medical Branch, Galveston, Texas, United States of America; Wayne State University, United States of America

## Abstract

Retinal Müller cells are major producers of inflammatory and angiogenic cytokines which contribute to diabetic retinopathy (DR). Over-activation of the Wnt/β-catenin pathway has been shown to play an important pathogenic role in DR. However, the roles of Müller cell-derived Wnt/β-catenin signaling in retinal neovascularization (NV) and DR remain undefined. In the present study, mice with conditional *β-catenin* knockout (KO) in Müller cells were generated and subjected to oxygen-induced retinopathy (OIR) and streptozotocin (STZ)-induced diabetes. Wnt signaling was evaluated by measuring levels of β-catenin and expression of its target genes using immunoblotting. Retinal vascular permeability was measured using Evans blue as a tracer. Retinal NV was visualized by angiography and quantified by counting pre-retinal nuclei. Retinal pericyte loss was evaluated using retinal trypsin digestion. Electroretinography was performed to examine visual function. No abnormalities were detected in the *β-catenin* KO mice under normal conditions. In OIR, retinal levels of β-catenin and VEGF were significantly lower in the *β-catenin* KO mice than in littermate controls. The KO mice also had decreased retinal NV and vascular leakage in the OIR model. In the STZ-induced diabetic model, disruption of β-catenin in Müller cells attenuated over-expression of inflammatory cytokines and ameliorated pericyte dropout in the retina. These findings suggest that Wnt signaling activation in Müller cells contributes to retinal NV, vascular leakage and inflammation and represents a potential therapeutic target for DR.

## Introduction

Retinal vascular leakage and retinal neovascularization (NV) are the major pathological changes leading to vision loss in diabetic retinopathy (DR) [1]. During the development of DR, retinal oxidative stress, inflammation and microvascular damages lead to blood-retinal barrier breakdown, capillary dropout and retina ischemia. Inflammation plays a prominent role in the development and progression of DR [2], [3]. Pro-inflammatory factors such as vascular endothelial growth factor (VEGF) and tumor necrosis factors-alpha (TNF-α) are upregulated in the retinae of patients with DR [4].

Recent studies have shown that the Wnt/β-catenin signaling pathway, which is known to regulate multiple biological and pathological processes including angiogenesis and inflammation, is involved in the pathogenesis of retinal vascular leakage, NV and inflammation in DR [5]–[10]. β-catenin, a transcription factor, acts as an essential effector in the Wnt signaling pathway. Upon binding of Wnt ligands to their receptors and co-receptors, the glycogen synthase kinase-3 (GSK-3) protein complex-mediated phosphorylation of β-catenin is inhibited, resulting in stabilization and accumulation of β-catenin in the cytosol. β-catenin then translocates to the nucleus, interacts with T cell factor (TCF)/lymphoid enhancer factor 1 (LEF-1) family of DNA-binding proteins, and activates transcription of numerous target genes including angiogenic and inflammatory factors, such as VEGF, TNF-α, and ICAM-1 which are implicated in DR [11], [12].

Müller cells are the principal glial cells of the retina and the major producer of inflammatory and angiogenic factors such as VEGF, TNF-α and ICAM-1 in DR. It has been shown that Müller cell-derived VEGF is a key contributor to retinal NV, retinal inflammation and vascular leakage in DR [13], [14]. These characteristics suggest that Müller cell dysfunction in diabetes may be relevant to the pathological processes of DR including vascular leakage and NV. However, the contribution of Wnt signaling activation in Müller cells to the development of retinal NV and DR has not been well defined. In the present study, we knocked *β-catenin* out in Müller cells to study the impacts of interrupted Wnt signaling in Müller cells on ischemia-induced retinal NV and retinal inflammation in DR.

## Materials and Methods

### Generation of Conditional β-catenin KO Mice

All animal procedures followed the Guidelines of the National Institutes of Health for the Use of Animal in Research and were approved by the Institutional Animal Care and Use Committees of the University of Oklahoma Health Sciences Center. The conditional KO mice were generated by cross-breeding β-catenin floxed mice with mice expressing Cre in retinal Müller cells. The Cre expressing mice were a gift from Dr. Yun Le at the University of Oklahoma Health Science Center. The cassettes of human PVMD2-rtTA and TRE-cre were used to generate the Cre transgenic mice. Cre expression in retinal Müller cells was confirmed by a Cre-activatable lacZ reporter mouse line (R26R) and a floxed interleukin six signal transducing receptor (gp130) mouse line, as described previously [15], [16]. Cre expression was induced by feeding pregnant mice with drinking water containing doxycycline at a dose of 2 mg/ml in a 5% sucrose solution from embryonic day 15 (E15) to postnatal day 1 (P1). The floxed β-catenin mice were purchased from the Jackson Laboratory. The knockout mice were genotyped by genomic PCR. Primers with sequences A (5′-AAG GTA GAG TGA TGA AAG TTG TT- 3′) and B (5′- CAC CAT GTC CTC TGT CTA TTC -3′) were used for PCR analysis to generate a 221-bp product for wild-type (WT) allele and a 324-bp product for *β–catenin* floxed allele, while primers C (5′- AGG TGT AGA GAA GGC ACT TAG C-3′) and D (5′-CTA ATC GCC ATC TTC CAG CAG G-3′) were used to detect a 411-bp product for *Cre* [16]. All WT mice were litter-mate flox/fox only controls, and received the same dose of Dox for the same duration as the KO mice. Mice were maintained in standard laboratory conditions with a 12-hour light/dark cycle.

### Ischemia-induced Retinal NV and DR

Oxygen-induced retinopathy (OIR) was generated by placing mice in 75% oxygen from P7 to P12, then returning them to room air, as described previously [17]. Diabetes was induced in mice by 5 daily intraperitoneal injections of streptozotocin (STZ, Sigma Chemical Co., St. Louis, MO) in 8-week-old male mice (weighing 20–25 g) after fasting for 8 h, at a dose of 55 mg/kg body weight in 10 mM citrate buffer (pH 4.5). Age-matched controls received injections of the citrate buffer. Diabetes was confirmed by measuring fasting blood glucose 3 days after the last injection of STZ and every 2 weeks thereafter. Mice with blood glucose levels greater than 350 mg/dL for at least 2 months were deemed diabetic (Table 1).

**Table 1:** Blood glucose of diabetic and non-diabetic mice.

Group		Blood glucose (mg/dl)	
Non-diabetic WT	164.1±5.6	161.5±4.7	164.8±6.4
Non-diabetic KO	168.3±4.2	165.4±6.8	169.4±7.8
Diabetic WT	375.2±15.5**	369.3±20.4**	373.5±16.8**
Diabetic KO	371.8±19.2**	374.7±17.6**	366.7±21.2**
Age	2 months	4 months	6 months

Data are mean±SD, n = 10–18, **p<0.01 compared with non-diabetic WT or KO mice.

### Primary Müller Cell Culture

Müller cell enrichment was conducted according to a previously established protocol [18]. Briefly, mice at P6-12 were euthanized, and the eyes were enucleated. The eyeballs were stored in Dubelcco’s Modified Eagles Medium (DMEM) containing 2 mM glutamine and 1/1000 penicillin/streptomycine, overnight in the dark. The eyeballs were then incubated in DMEM containing 0.1X trypsin and 70 U/mL collagenase I, at 37°C for 1 hr. The retina was then carefully dissected, avoiding contamination from the retinal pigment epithelium (RPE) and vascular cells, and dissociated into small pieces (∼1 mm^2^). The fragments were cultured in DMEM with 10% fetal bovine serum (FBS) and 1/100 penicillin/streptomycin. The retinal aggregates and debris were then removed. Attached cells were trypsinized and cultured in DMEM containing 10% FBS for another 5 days to obtain a purified population. Cultured cells of the 4^th^–6^th^ passages were utilized, and serum free DMEM medium was applied before further analysis.

### Adenovirus infection

Primary Müller cells were infected with an adenovirus expressing Cre (Vector BioLabs, Ad-Cre) for 48 hr. An adenovirus expressing GFP (Ad-GFP) was used as control.

### Western Blot Analysis

Western blot analysis was performed as described previously [19]. Mouse anti-β-actin monoclonal antibody (1∶5000) and rabbit anti-TNF-α polyclonal antibody (1∶500) were purchased from Abcam (Cambridge, MA). Rabbit anti-β-catenin antibody (1∶2000), rabbit anti-VEGF antibody (1∶1000) and mouse anti-ICAM-1 antibody (1∶500) were purchased from Santa Cruz Biotechnology (Santa Cruz, CA). Horseradish peroxidase-labeled secondary antibodies (1∶2000 dilution, Santa Cruz Biotechnology) were used for immunoblotting. Densitometry was performed using ImageJ software (Wayne Rasband, National Institutes of Health, Bethesda, MD) and normalized by β -actin levels.

### Immunofluorence staining

Immunofluorence staining was performed according to a procedure described previously [13]. Briefly, cultured cells were fixed and co-stained with an anti-glutamine synthetase (GS) antibody (1∶10000 dilution, Abcam) and an anti-β-catenin antibody (1∶1000 dilution, Santa Cruz). Retinal sections (4-µm) were blocked with 10% goat serum, 3% BSA and 0.25% Triton X-100, and then incubated with an anti-β-catenin antibody (1∶100 dilution) and an anti-GS antibody (1∶1000 dilution).

### Vascular Permeability Assay

Retinal vascular permeability was measured using Evans blue as tracer following a documented procedure [20]. Evans blue leakage was calculated using the following equation: [retinal Evans blue concentration (µg/ml)/retinal weight (mg)]/[blood EB blue concentration (µg/ml)×circulation time (h)].

### Retinal Angiography

Retinal angiography was performed following a documented procedure with minor modifications [21]. The non-perfused area in flat-mounted retinas was measured using SPOT software (Diagnostic Instruments, Sterling Heights, MI).

### Retinal Morphology Examination

Retinal morphometric analysis was performed as described previously [22]. Briefly, retinal sections (5 µm in thickness) through the optic nerve were defined as central and stained with haematoxylin and eosin (H&E) and examined under a light microscope. The total thickness of the neural retina and the thickness of the different retinal layers were measured at the same locations of the central retina in each eye.

### Quantification of Pre-Retinal Neovascularization

Pre-retinal NV was quantified as described previously [23]. Non-continuous 5-µm retina sections were stained with H&E and examined by masked observers using light microscopy. Nuclei extended to the vitreal side of the inner limiting membrane were counted as pre-retinal vascular cells.

### Retinal Trypsin Digestion Assay

Retinal trypsin digestion assay was performed according to a previously published protocol [24]. The eyes were enucleated and fixed in 4% paraformaldehyde for at least 24 hr. The eyes were equatorially bisected and the entire retina was removed. The retinas were washed overnight in distilled water and incubated with 3% trypsin (Difco Trypsin 250, Becton Dickinson and Company, Sparks, MD) in 0.1 M Tris, 0.1 M maleic acid containing 0.2 M NaF for 1 hr at 37°C. Non-vascular tissues were carefully brushed away following completion of the digestion. Retinal vasculature was then transferred and flat-mounted onto clean glass slides for periodic acid-Schiff (PAS) and hemotoxylin staining. Six random mid-retina areas (between the center and periphery of the retina) were used to count retinal NV tufts, pericytes (PC) and endothelial cells (EC) under a light microscope. Only retinal capillaries were included in the cell count. Nuclear morphology was used to distinguish PC from EC. The ratio of EC to PC was determined by counting respective nuclei under a microscope at a magnification of 400.

### Electroretinogram (ERG) Recording

ERG was performed as described previously [25]. For quantitative analysis, the B-wave amplitude was measured between the peaks of A- and B-waves.

### Statistical Analysis

The quantitative data were analyzed and compared with the littermate control. Quantitative data was expressed as mean ± SEM or mean ± SD. Statistical difference was analyzed by Student’s t test with statistical significance difference set at p<0.05.

## Results

### Evaluation of *β-catenin* KO efficiency in primary retinal Müller cells from the conditional *β-catenin* KO mice

**Figure 1:**
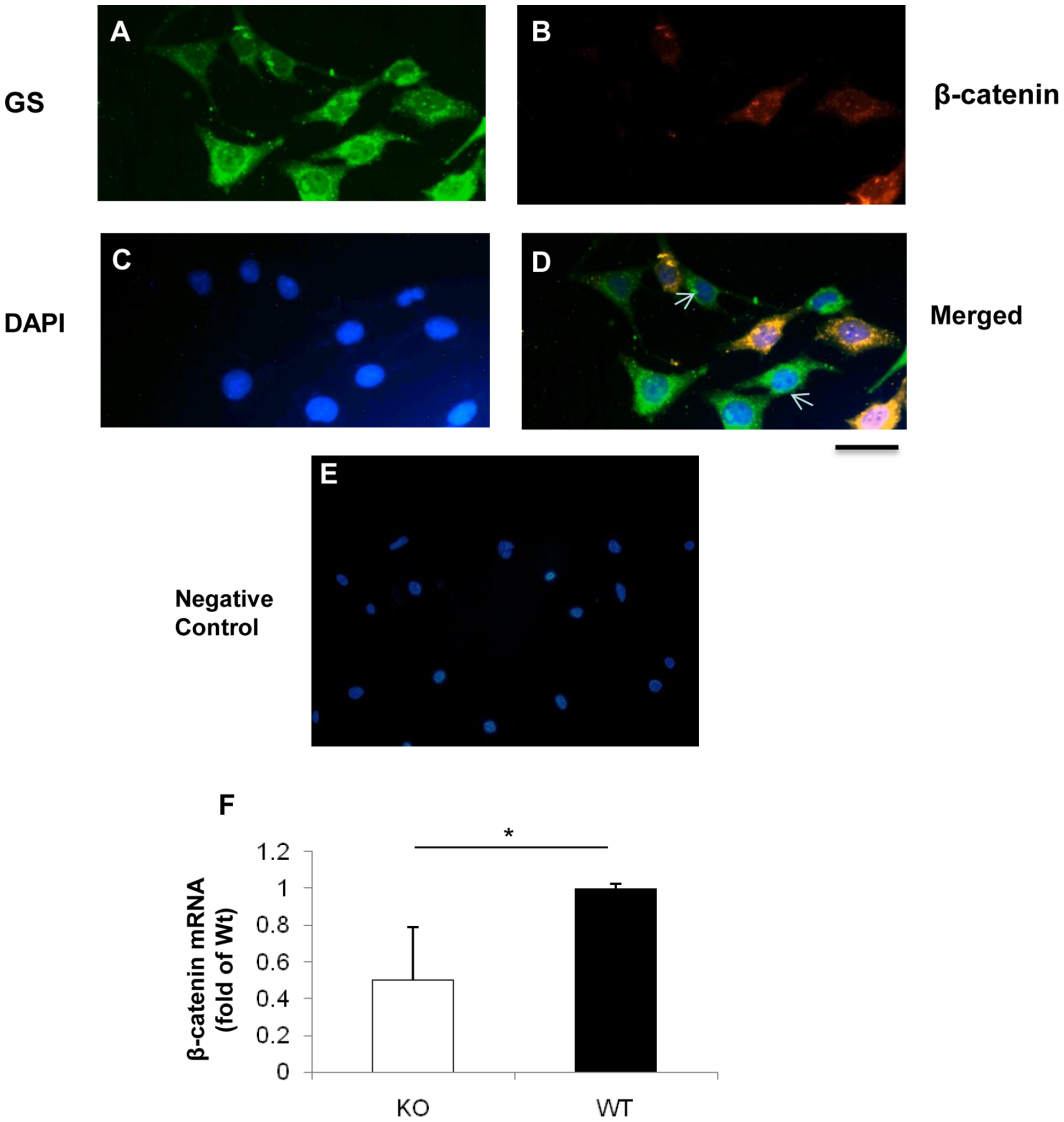
Immunostaining of primary Müller cells isolated from the conditional *β-catenin* KO mice. (**A–E**) Primary Müller cells from the conditional *β-catenin* KO mice were co-immunostained with an antibody against GS (green) (**A**) and an antibody for β-catenin (red) (**B**). The nuclei were counterstained with 4′,6-diamidino-2-phenylindole (DAPI, blue **C**). Immunostaining of GS (green) and β-catenin (red) in primary Müller cells from the β-catenin KO mice merged with nuclei stained with DAPI (blue) (**D**). Negative IgG is used as control (**E**). Arrows indicate Müller cells lacking β-catenin expression. Scale bar: 50 µm. Real-time PCR analysis of β-catenin mRNA levels in the primary Müller cells (**F**). Values are mean±SEM; n = 3; *p<0.05.

To assess the efficiency of *β-catenin* disruption in the *β-catenin* KO mice, primary Müller cells were isolated and cultured from the retina of newborn *β-catenin* KO mice. Immunostaining of GS, a Müller cell marker, showed that more than 90% of the isolated primary cells were GS-positive (Fig. 1A, B). Some of the GS-positive primary Müller cells demonstrated diminished β-catenin levels (Fig. 1C, D). Furthermore, real-time PCR analysis showed significantly decreased β-catenin mRNA levels in the primary Müller cells (Fig. 1F).

**Figure 2:**
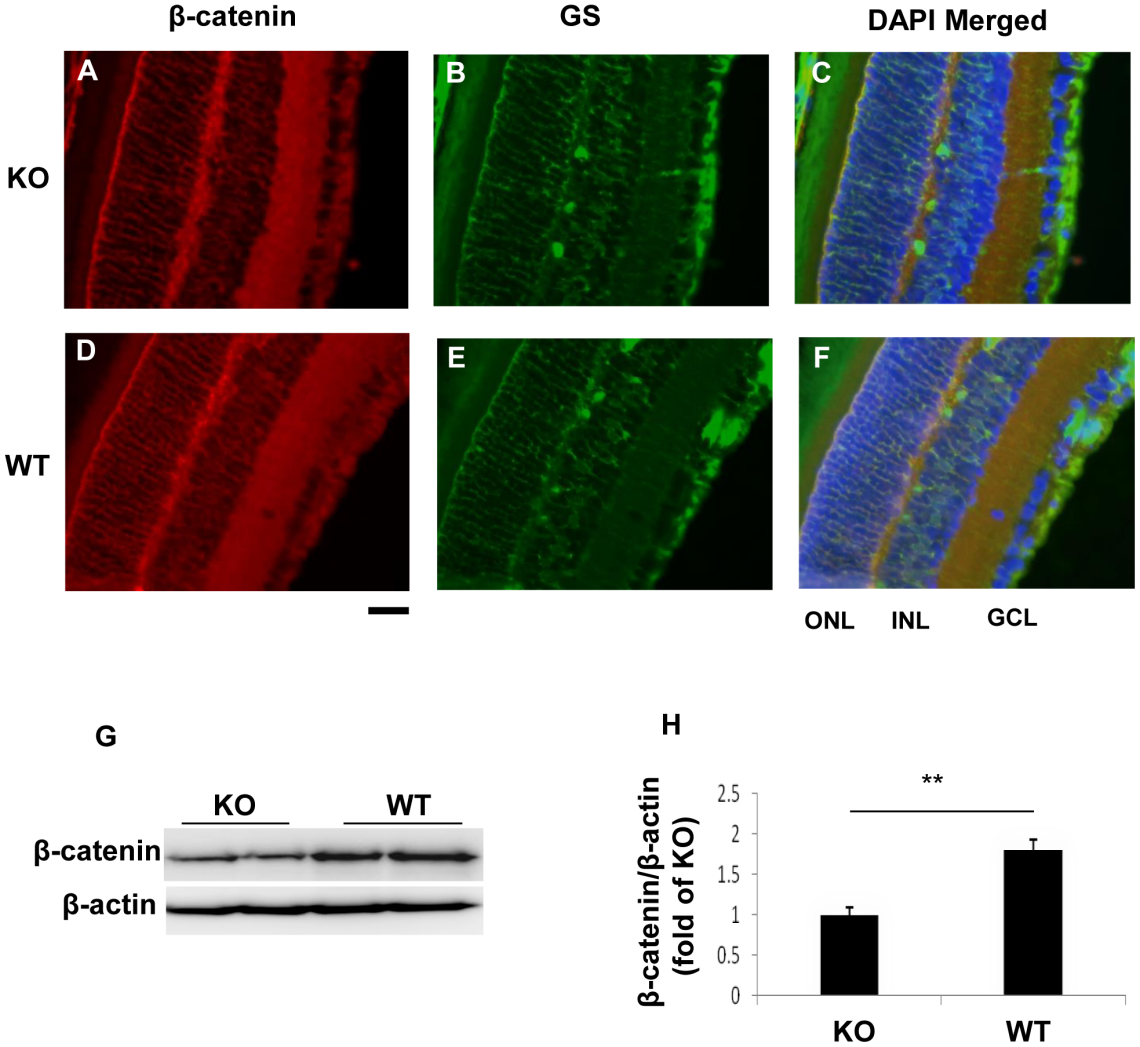
Decreased β-catenin levels in the retinas of the diabetic and OIR KO mice. (**A–H**) Immunostaining of β-catenin (red) and GS (green) in the retina of the diabetic *β-catenin* KO mice (**A–C**) and WT mice (**D–F**), with the nuclei counterstained with DAPI (blue). Scale bar: 50 µm. ONL, outer nuclear layer; INL, inner nuclear layer; GCL, ganglion cell layer. (**G, H**) Western blot analysis of β-catenin in the retina of OIR mice at P17 (**G**) and quantification by densitometry (**H**). Values are mean±SEM; n = 6; **p<0.01.

To further verify the efficiency of *β-catenin* KO in the retina, we measured β-catenin levels in the retina of diabetic and OIR mice. Immunostaining showed decreases of β-catenin immunosignals in the inner retina of the diabetic *β-catenin* KO mice, compared with diabetic WT mice (Fig. 2A–F). Western blot analysis using the retina homogenate showed approximately a 2-fold decrease of β-catenin levels in the retina of the OIR KO mice (Fig. 2G, H).

### Lack of retinal phenotypes in conditional *β-catenin* KO mice under normal conditions

**Figure 3:**
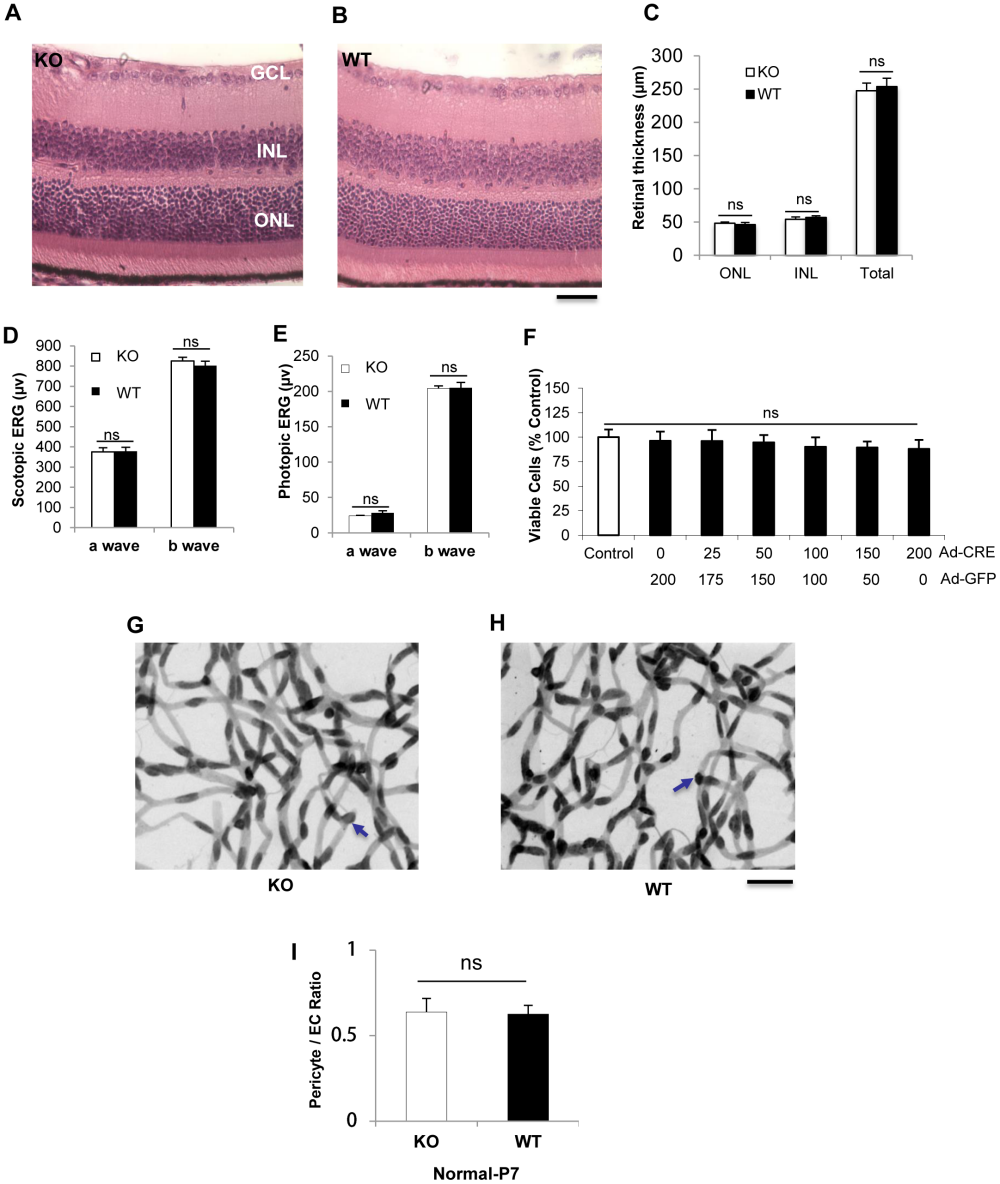
Phenotypes of the β-catenin KO mice in normal conditions. Representative retinal sections of 20-week-old *β-catenin* KO (KO) (**A**) and WT (**B**) mice stained with H&E. ONL, outer nuclear layer; INL, inner nuclear layer; GCL, ganglion cell layer. Scale bar: 50 µm. (**C**) Quantification of thicknesses of the retina, ONL and INL (n = 5). (**D, E**) Scotopic (**D**) and photopic (**E**) ERG were recorded at 12 weeks (n = 10). The amplitudes of a and b waves were measured. (**F**) Primary Müller cells from *β-catenin* floxed mice were infected with adenovirus expressing Cre for 48 hr. An adenovirus expressing GFP was used as control virus. Viable cells were quantified by MTT and expressed as % of the control (n = 3). (**G, H**) Retina trypsin digestion of mice at P7. Blue arrows indicate pericyte nuclei. Scale bar, 50 µm. (**I**) Quantification and statistical analysis of pericyte/endothelial cell (EC) ratio. (Values are mean±SEM (ns, not significant).

To determine whether the loss of Müller cell-derived β-catenin in the *β-catenin* KO mice affects retinal development, retinal morphology, vasculature and visual function were examined and compared between the KO mice and the age-matched WT controls under normal conditions. Retinal morphology was examined in retinal sections stained with H&E in 20 week-old mice. Retinal thickness was measured using morphometry. No significant differences were observed in the thicknesses of the outer nuclear layer, inner nuclear layer and entire retina between the conditional KO mice and age-matched WT mice (Fig. 3A–C). Retinal visual function evaluated by ERG recording showed no significant differences in the amplitudes of a or b waves in rod and cone ERG responses between the conditional KO mice and WT mice at age of 12 weeks (Fig. 3D–E). To investigate whether the deficiency of β-catenin affected Müller cell viability, primary Müller cells from β-catenin flox/flox mice were infected with Ad-Cre for 48 hr. Cell viability was measured by MTT assay. β-catenin KO did not affect the viability of Müller cells (Fig. 3F). To evaluate pericyte and endothelial cell density under normal conditions, trypsin digestion of the retina was performed on KO and WT mice at P7. There was no significant difference in the ratio of pericyte/endothelial cells between WT and KO mice (Fig. 3G–I). These data suggested that deficiency of β-catenin did not affect retinal development and Müller cell viability under normal conditions.

### Ameliorated ischemia-induced retinal NV and vascular leakage in the *β-catenin KO* OIR mice

**Figure 4:**
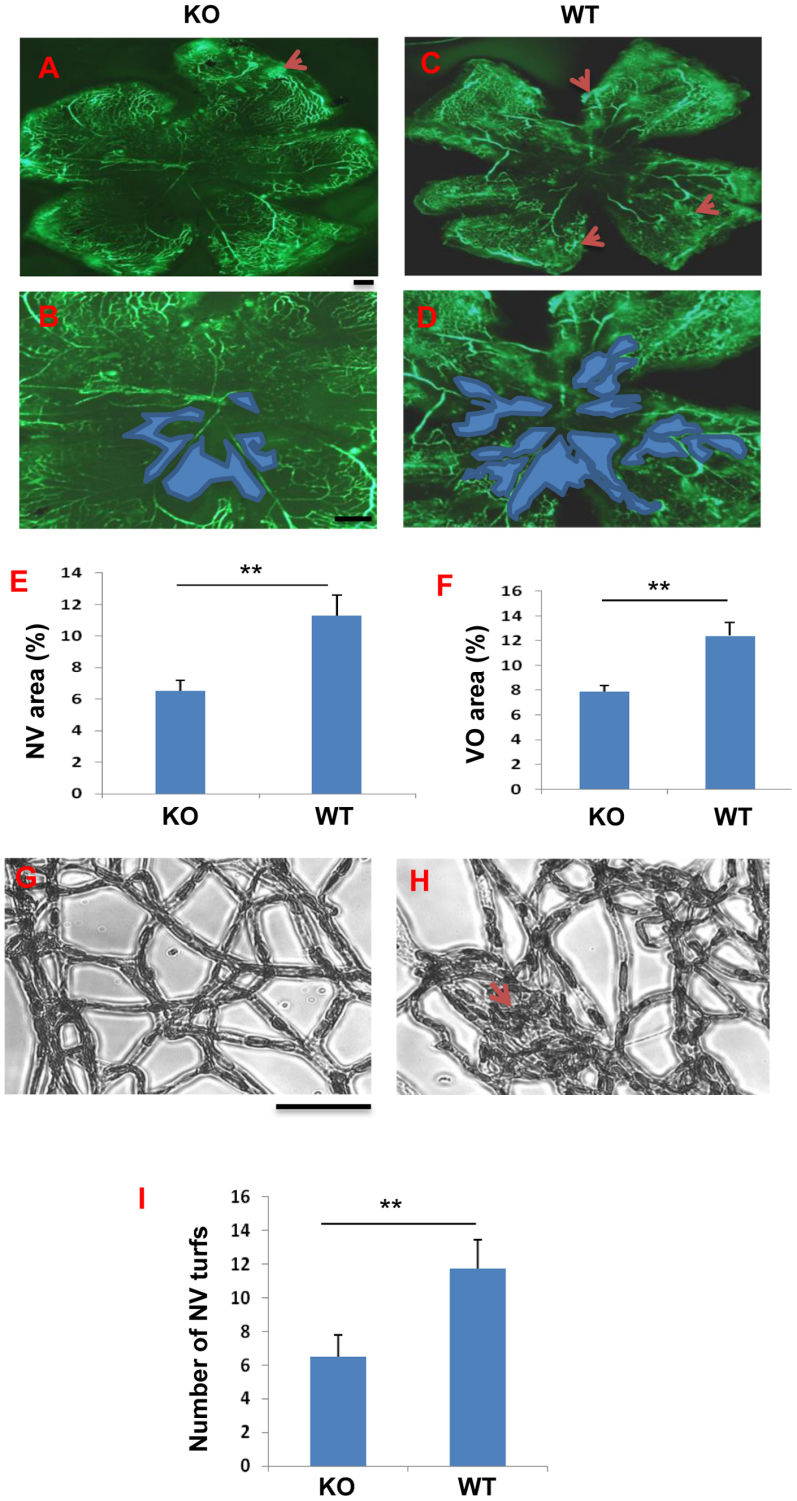
Amelioration of ischemia-induced retinal NV in the *β-catenin* KO mice with OIR. (**A–E**) Representative fluorescein angiographs of the *β-catenin* KO (KO) (**A, B**) and WT (**C, D**) mice at age P17. Red arrows in **A** and **C** indicate NV areas. Blue areas in (**B, D**) indicate vaso-obliteration (VO) regions in the central retina that was used in the quantification. (**E**) Quantification of the retinal NV area in fluorescein angiographs (n = 10). (**F**) Quantification of the VO area (n = 10). (**G, H**) Visualization of NV turfs by retina trypsin digest assay. Scale bar, 50 µm. (**I**) Quantification of retinal NV turfs in the retina with trypsin digest. All values are mean±SEM (n = 5, **p<0.01).

**Figure 5:**
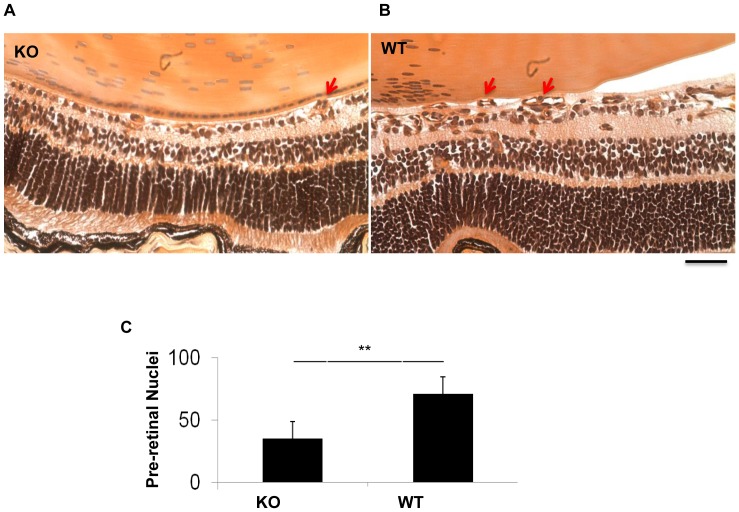
Analysis of OIR-induced pre-retinal NV. (**A, B**) Representative H&E-stained retinal sections showing pre-retinal NV cells in the *β-catenin* KO (**A**) and WT (**B**) mice with OIR at age of P17. Arrows indicate vascular nuclei in the vitreous. (**C**) Quantification and statistical analysis of pre-retinal vascular nuclei (mean±SEM, n = 5, **p<0.01).

To evaluate the role of Müller cell-derived β-catenin in ischemia-induced retinal NV, the *β-catenin* KO mice and age-matched WT mice were subjected to OIR from P7 to P12. As shown by retinal angiography at P17, the *β-catenin* KO/OIR mice developed smaller ischemia-induced retinal NV areas and non-perfused areas in the flat-mounted retina, compared to WT/OIR mice (Fig. 4A–F). Retinal vasculature after trypsin digestion showed fewer retinal NV tufts in *β-catenin* KO/OIR mice, compared to WT/OIR mice (Fig. 4G–I). To quantify pre-retinal NV, vascular nuclei anterior to the inner limiting membrane were counted in the cross sections of the retina at P17. The conditional *β-catenin* KO/OIR mice showed significantly fewer pre-retinal neovascular cells (36% of WT), compared to WT/OIR mice under the same conditions (Fig. 5).

**Figure 6:**
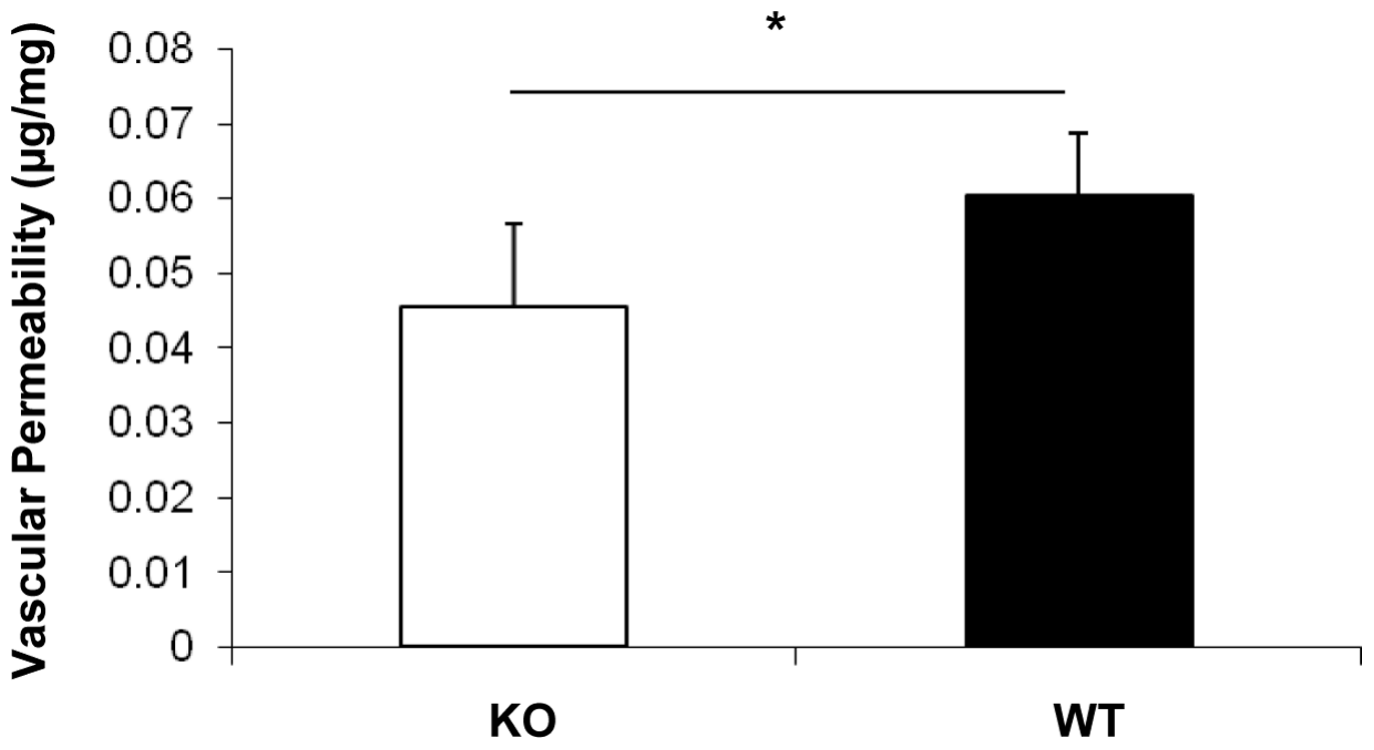
Reduced OIR-induced retinal vascular leakage in the *β-catenin* KO mice. Retinal vascular leakage was quantified by vascular permeability assay using Evans blue dye as a tracer. Extravasation of Evans blue dye in the mouse retinas of the KO and WT mice was quantified and normalized by total retinal protein concentration (Mean±SD, n = 5, *p<0.05).

To quantify vascular leakage, Evans blue was used as tracer for a vascular permeability assay. OIR induced significant less retinal vascular leakage at P17 in the *β-catenin* KO mice, compared with WT mice (Fig. 6).

### Attenuated diabetes-induced retinal pericyte loss in the conditional *β-catenin* KO mice

**Figure 7:**
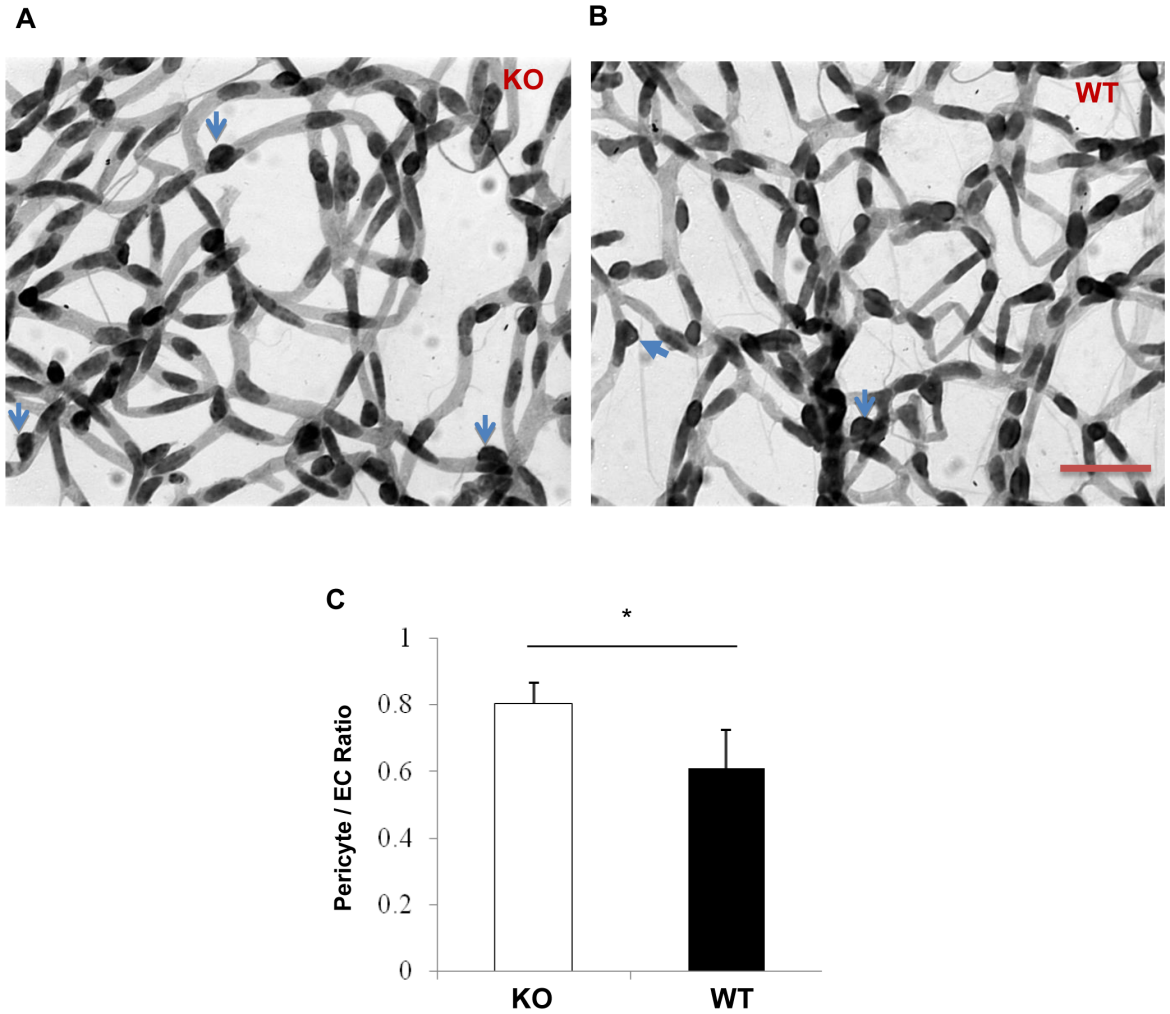
Retina trypsin digestion analysis in the *β-catenin* KO and WT mice with diabetes. (**A, B**) Retina trypsin digestion assay was performed in the *β-catenin* KO mice (**A**) and WT mice (**B**) with 4 months of STZ-induced diabetes. Blue arrows indicate pericyte nuclei. Scale bar, 50 µm. (**C**) Quantification and statistical analysis of pericyte/endothelial cell (EC) ratio (mean±SEM, n = 5, *p<0.05).

Pericyte loss is a common pathological feature at the early stage of DR and is believed to be associated with retinal inflammation [26]. To determine whether Müller cell-derived β-catenin contributes to the diabetes-induced retinal inflammation and pericyte degeneration, the retina vasculature was examined using the trypsin digestion assay. At 4 months after the onset of STZ-induced diabetes, the diabetic *β-catenin* KO mice showed a significantly higher pericyte/endothelial cell ratio than the diabetic WT mice with similar levels of hyperglycemia, suggesting ameliorated pericyte loss in diabetes (Fig. 7). The diabetic *β-catenin* KO mice and WT mice were have similar levels of hyperglycemia (Table 1).

### Suppressed over-expression of VEGF and inflammatory factors in the retinas of the *β-catenin* KO mice

**Figure 8:**
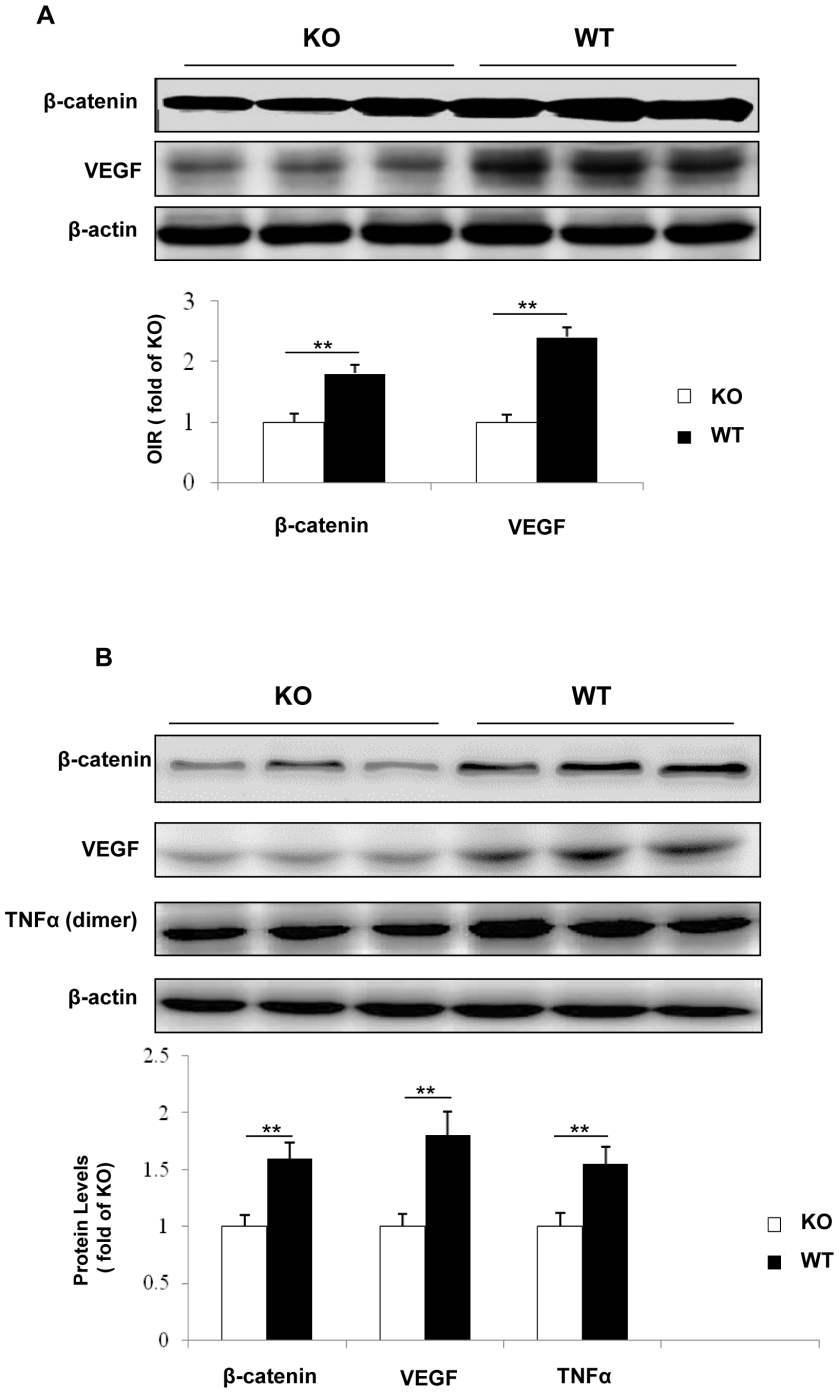
Western blot analysis for β-catenin and its target gene expression in the retinas of OIR and STZ-induced diabetic mice. (**A**) β-catenin and VEGF levels were measured by Western blot analysis in the retina from the *β-catenin* KO (KO) mice and WT controls with OIR at P17 and semi-quantified with densitometry. (**B**) Western blot analysis of β-catenin, VEGF and TNF-α in the retinas from the KO mice and WT controls 4 months after the onset of STZ-induced diabetes. Values are mean±SEM (n = 6, **p<0.01).

In DR, VEGF, a target gene regulated by β-catenin, is a major angiogenic and inflammatory factor that induces retinal NV and vascular leakage [27], [28]. To evaluate the impacts of disruption of *β-catenin* in Müller cells on the over-expression of VEGF, levels of VEGF in the retinas of OIR mice and STZ-diabetic mice were determined by Western blot analysis. Under the same conditions, Western blot analysis showed that VEGF expression in the retina of WT mice with OIR was almost 2.5-fold higher than that in the *β-catenin* KO mice with OIR (Fig. 8A). VEGF expression in the retina of STZ-induced WT diabetic mice was almost 2-fold higher than that in the diabetic *β-catenin* KO mice (Fig. 8B).

Diabetes-induced over-expression of inflammatory factors, such as TNF-α, is known to play important roles in DR [13]. To investigate the role of β-catenin in Müller cells in the over-expression of these inflammatory factors, TNF-α levels were measured in the retina of the *β-catenin* KO mice and WT mice with 4 months of STZ-induced diabetes. The diabetic WT group exhibited elevated TNF-α levels in the retina, approximately 1.5-fold higher than the *β-catenin* KO mice with the same degree and duration of hyperglycemia. Meanwhile, expression of a constitutive active mutant of β-catenin up-regulated the expression of VEGF and TNF-α in primary *β-catenin* KO Müller cells (Fig. S1). These data suggested that β-catenin in Müller cells is a major pathological mediator of retinal inflammation in diabetes.

## Discussion

Retinal inflammation is believed to be a key pathogenic process leading to pericyte loss, vascular leakage and NV in DR [29]. Retinal Müller cells play an important role in retinal inflammation in DR. Wnt/β-catenin signaling is known to regulate expression of multiple pro-inflammatory and pro-angiogenic factors [30]. The Wnt/β-catenin pathway is activated in the retinas of diabetic models and in the retina with ischemia-induced retinal NV [31]. The present study used conditional KO of *β-catenin*, a key effector of the canonical Wnt pathway, to demonstrate that interruption of Wnt signaling in retinal Müller cells attenuates ischemia-induced retinal NV and vascular leakage in the OIR model. In the STZ-induced diabetic model, *β-catenin* KO also ameliorated diabetes-induced retinal inflammation and pericyte loss. These results suggest that Wnt signaling in Müller cells plays a central role in inflammation and NV in DR.

Our previous studies showed that Wnt signaling activation in DR occurs mainly in the inner retina, indicating the location of the Wnt/β-catenin pathway activation during DR [7]. Müller cells form architectural supporting structures stretching radially across the thickness of the retina. They regulate retinal homeostasis and interact with nearly all retinal cell types. Müller cells are a major cell type in the inner retina, and serve as the main source of angiogenic and inflammatory factors, and thus, Müller cells have been suggested to be a major contributor to DR [14], [15]. To address whether the Wnt/β-catenin pathway activation in Müller cells is essential for the over-production of pro-angiogenic and pro-inflammatory factors in DR, we established a Müller cell-specific *β-catenin* KO mice model using the Cre/lox system. Similar to the phenotypes in *VEGF* and *HIF-1α* KO in Müller cells reported previously [13], [15], *β-catenin* KO in Müller cells also attenuated over-expression of VEGF and TNF-α, reduced ischemia-induced retinal vascular leakage and NV, suggesting that Wnt signaling activation in Müller cells is responsible, at least in part, for VEGF and TNF-α over-production in Müller cells in DR.

Global *β-catenin* KO is embryonic lethal, suggesting that β-catenin is essential for development [32]. To determine if *β-catenin* KO in Müller cells affects retinal development and function, we examined the structure and function of the retina. Histological analysis and ERG recording showed that postnatal disruption of *β-catenin* in Müller cells presented no detectable deleterious phenotypes in normal conditions, suggesting that the postnatal loss of Müller cell-derived β-catenin did not disturb the normal retinal structure and vision under normal conditions. It is likely that there may be other signaling pathways to compensate for the interruption of the Wnt/β-catenin pathway in Müller cells. Unlike global gene KO, the efficiency of *β-catenin* KO using the Cre/Lox system is approximately 50%. Most of these conditional gene knockout studies are not expected to have high efficiency of Cre-mediated recombination because of the limitations of tetracycline-inducible gene expression technology. It is also possible that the remaining β-catenin levels in Müller cells of the KO mice may be sufficient for the retinal development and function.

Despite the lack of phenotype in the *β-catenin* KO mice under normal conditions, the KO mice displayed less severe pathological phenotypes under retinal ischemia and diabetes. OIR is a model of ischemia-induced retinal NV commonly used to study proliferative DR [33]. Its pathology of pre-retinal NV and pathogenesis such as ischemia-induced over-expression of VEGF are similar to those of proliferative DR. In the OIR model, WT mice showed accumulation of β-catenin, over-expression of VEGF in the retina, and developed severe vascular leakage and pre-retinal NV, consistent with our previous studies [13], [25]. In contrast, the *β-catenin* KO mice with OIR demonstrated attenuated expression of VEGF and reduced ischemia-induced vascular leakage, decreased retinal neovascular tufts and pre-retinal vascular cells in comparison to WT OIR mice. These observations indicate that Wnt signaling in Müller cells is a key contributor to ischemia-induced VEGF over-expression, vascular leakage and NV in the OIR model.

Our previous studies demonstrated that Wnt signaling is activated in the retina of diabetic models [7]–[9]. To establish the pathogenic role of the Wnt/β-catenin pathway activation in DR, we induced diabetes in the *β-catenin* KO mice. In congruence with the decreased β-catenin levels in the retina of the diabetic KO mice, retinal VEGF and TNF-α levels were also significantly lower in the retina of the diabetic KO mice, compared with that in the WT mice with the same duration of diabetes and similar levels of hyperglycemia (Table 1). These results showed that disruption of the *β-catenin* gene in retinal Müller cells alleviated retinal inflammation induced by diabetes, indicating that Müller cell-derived β-catenin is an important contributor to retinal inflammation in DR.

Pericytes contribute to vascular stability and control endothelial cell proliferation [34]. Pericyte loss is a main characteristic of early stages of DR. To determine whether Müller cell-derived β-catenin contributes to diabetes-induced retinal inflammation, capillary pericyte degeneration was investigated using a trypsin digestion assay. Diabetic *β-catenin* KO mice showed ameliorated pericyte loss in comparison to WT controls with the same duration of diabetes. As inflammation is believed to play a key role in pericyte loss in diabetes, decreased pericyte dropout in the *β-catenin* KO mice provides further evidence that Wnt signaling in Müller cells plays a crucial role in retinal inflammation in DR.

Although there are some effective sight-saving therapies such as laser photocoagulation and vitrectomy for DR [35], these are invasive and associated with significant negative side effects. Recently, anti-VEGF strategies have achieved impressive therapeutic effects in some DR patients. However, these anti-VEGF compounds are not effective in all DR patients, and provide only temporary alleviation of DR. New therapeutic targets need to be defined for the development of new treatments of DR. Because β-catenin is an essential effector for Wnt signaling and regulates multiple inflammatory and angiogenic factors, β-catenin in retinal Müller cells represents a promising drug target for simultaneous suppression of multiple inflammatory and angiogenic factors in diabetes.

The Müller cell-derived *β-catenin* KO method employed in this study does not disrupt the gene in every targeted cell. The Cre/lox system requires doxycyclin induction, and the Cre-mediated recombination does not occur in all of Müller cells. Thus, these may account for the incomplete *β-catenin* KO in the retinal Müller cells. Although the KO was not complete, significant impacts of the *β-catenin* KO were nevertheless observed under the stress conditions. Future studies are needed to improve the efficiency of *β-catenin* KO in Müller cells to evaluate the beneficial and adverse effects. The long-term impacts of interruption of Wnt signaling in Müller cells on vision remains to be evaluated in the future.

DR is a chronic diabetic complication related to multiple pathogenic mechanisms [36]. Although we have shown that Müller cell-derived β-catenin is an important pathogenic factor of DR, our results do not exclude other cell types and pathways that are also involved. In addition to Müller cells, activation of Wnt signaling in the RPE and capillary endothelial cells can also play important roles in the development of DR [37]–[39]. VEGF, ICAM-1 and other contributing factors are also regulated by other mechanisms, such as HIF-1α and oxidative stress [14]. Although the Wnt/β-catenin pathway has cross-talks with other pathways such as oxidative stress and HIF-1α [16], the underlying mechanism remains unclear. Many other contributing pathways such as the polyol pathway, nonenzymatic protein glycation, protein kinase C (PKC) activation and the renin-angiotensin-aldosterone system have been extensively studied [40]–[42]. The conditional *β-catenin* KO mice may become a useful tool to investigate interactions between these signaling pathways in DR.

## Supporting Information

Figure S1 **Restoring β-catenin Induced Expression of Inflammatory Factors in Retina Müller Cells.** Primary Müller Cells were infected with Ad-GFP and Ad-S37A at the same MOI for 48 hours. Levels of β-catenin, VEGF and TNF-α (**A**) were determined by Western blot analysis using 50 µg total protein. Quantification of β-catenin, VEGF and TNF-α (**B**) by densitometry, normalized by β-actin levels and expressed as percentage of the control. All values are mean ± SD (n = 3). *p<0.05.
(TIF)
