# Supplementary figures and images for: Interruption of Wnt Signaling in Müller Cells Ameliorates Ischemia-Induced Retinal Neovascularization
Source: PLoS One. 2014 Oct 1;9(10):e108454. doi: 10.1371/journal.pone.0108454 (PMC4182699; doi:10.1371/journal.pone.0108454)

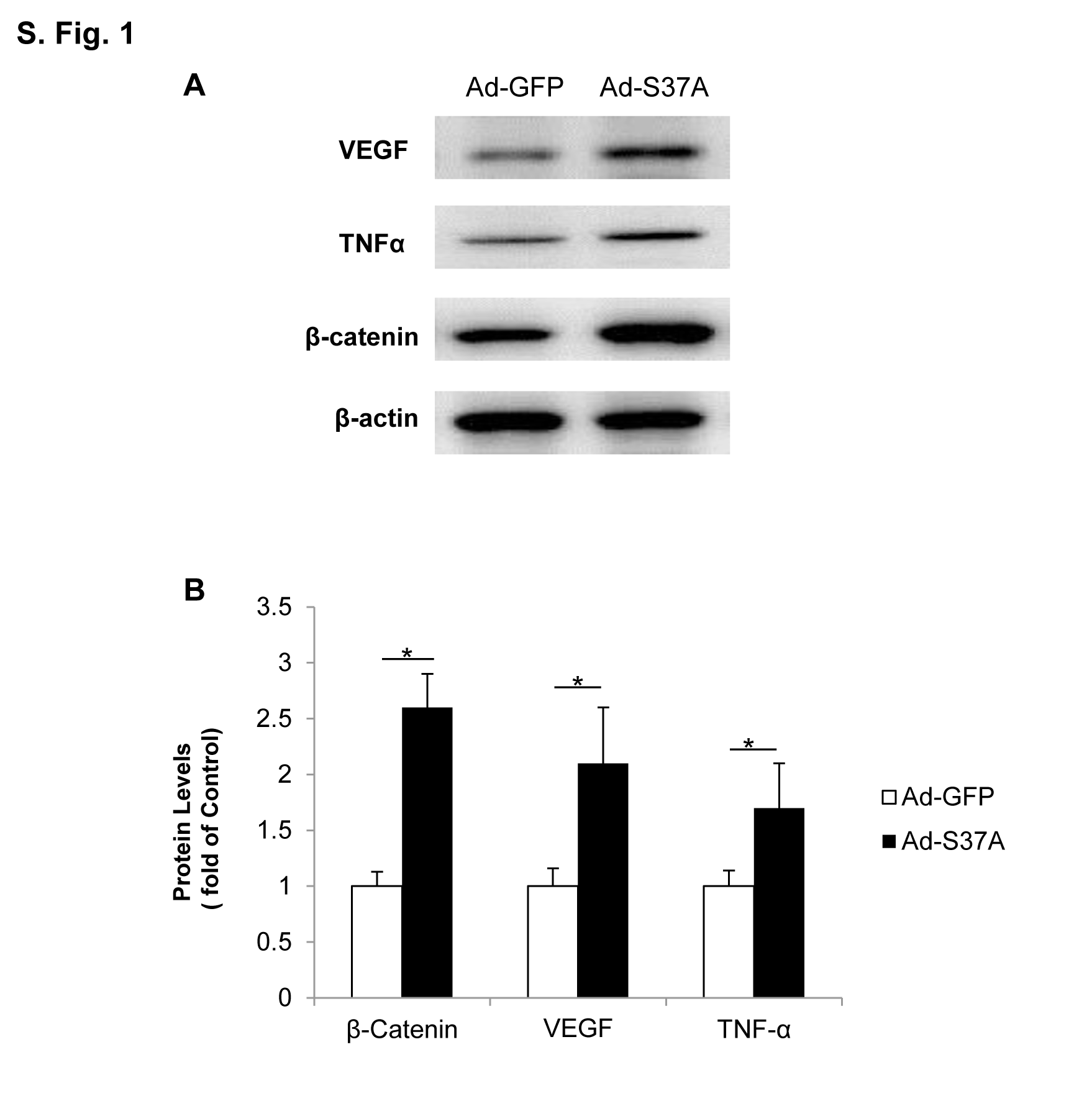

Supplement: Figure S1 — Restoring β-catenin Induced Expression of Inflammatory Factors in Retina Müller Cells. Primary Müller Cells were infected with Ad-GFP and Ad-S37A at the same MOI for 48 hours. Levels of β-catenin, VEGF and TNF-α (A) were determined by Western blot analysis using 50 µg total protein. Quantification of β-catenin, VEGF and TNF-α (B) by densitometry, normalized by β-actin levels and expressed as percentage of the control. All values are mean ± SD (n = 3). *p<0.05. (TIF) [file pone.0108454.s001.tif]
